# The AC Soft Magnetic Properties of FeCoNi*_x_*CuAl (1.0 ≤ *x* ≤ 1.75) High-Entropy Alloys

**DOI:** 10.3390/ma12244222

**Published:** 2019-12-16

**Authors:** Zhongyuan Wu, Chenxu Wang, Yin Zhang, Xiaomeng Feng, Yong Gu, Zhong Li, Huisheng Jiao, Xiaohua Tan, Hui Xu

**Affiliations:** 1 Institute of Materials, School of Materials Science and Engineering, Shanghai University, Shanghai 200072, China; wzy951122@gmail.com (Z.W.); wcx123581@163.com (C.W.); zhangyin0208@163.com (Y.Z.); fengxiaomeng96@163.com (X.F.); gy2018@hznu.edu.cn (Y.G.); 2 Qianjiang College, Hangzhou Normal University, Hangzhou 310036, China; 3 College of Materials and Environmental Engineering, Institute for Advanced Magnetic Materials, Hangzhou Dianzi University, Hangzhou 310018, China; hanying880205@163.com; 4 Tescan China, Ltd., Shanghai 201112, China; Huisheng.Jiao@tescanchina.com

**Keywords:** high-entropy alloys, Ni content, AC soft magnetic properties, AC hysteresis loss (*P_h_*), eddy current loss (*P_e_*)

## Abstract

High-entropy alloys (HEAs) with soft magnetic properties are one of the new candidate soft magnetic materials which are usually used under an alternating current (AC) magnetic field. In this work, the AC soft magnetic properties are investigated for FeCoNi*_x_*CuAl (1.0 ≤ *x* ≤ 1.75) HEAs. The X-ray diffraction (XRD) and scanning electron microscope (SEM) show that the alloy consists of two phases, namely a face-centred cubic (FCC) phase and a body-centred cubic (BCC) phase. With increasing Ni content, the FCC phase content increased. Further research shows that the AC soft magnetic properties of these alloys are closely related to their phase constitution. Increasing the FCC phase content contributes to a decrease in the values of AC remanence (*AC B_r_*), AC coercivity (*AC H_c_*) and AC total loss (*P_s_*), while it is harmful to the AC maximum magnetic flux density (*AC B_m_*). *P_s_* can be divided into two parts: AC hysteresis loss (*P_h_*) and eddy current loss (*P_e_*). With increasing frequency *f*, the ratio of *P_h_*/*P_s_* decreases for all samples. When *f* ≤ 150 Hz, *P_h_*/*P_s_* > 70%, which means that *P_h_* mainly contributes to *P_s_*. When *f* ≥ 800 Hz, *P_h_*/*P_s_* < 40% (except for the *x* = 1.0 sample), which means that *P_e_* mainly contributes to *P_s_*. At the same frequency, the ratio of *P_h_*/*P_s_* decreases gradually with increasing FCC phase content. The values of *P_e_* and *P_h_* are mainly related to the electrical resistivity (*ρ*) and the *AC H_c_*, respectively. This provides a direction to reduce *P_s_*.

## 1. Introduction

Conventional alloys are usually based on a certain metallic element, adding a few other elements to improve the property or fine tune the performance according to different application requirements [1,2]. In 2004, a new class of alloys was put forward by Cantor [3] and Yeh [4], namely high-entropy alloys (HEAs). These alloys contain five or more equiatomic or near-equiatomic ratio elements, each of which has an atomic percentage between 5% and 35%. The HEAs are conducive to the formation of a solid solution phase, typically with a structure of a body-centred cubic (BCC) [5,6], a face-centred cubic (FCC) [7,8,9], a hexagonal stacked (HCP) [10,11], or a mixture of the above mentioned structures [12,13,14]. In the past decade, HEAs have drawn extensive attention because of their excellent mechanical and chemical properties, such as great thermal stability [15], good corrosion resistance [16,17], good wear resistance [18], excellent strength [19] and high hardness [20].

Some HEAs show soft magnetic properties due to their ferromagnetic elements such as Fe, Co and Ni [21,22,23,24,25]. Zuo et al. [26] showed that the CoNiMnGa HEAs had a high saturation magnetisation (*M_s_*) of about 115.92 emu/g, and a low coercivity (*H_c_*) of about 27.9 Oe. Liu et al. [27] found that FeCoNi_1.5_CuAl HEA not only had good mechanical properties (maximum compressive strength *σ_max_* = 1725 MPa), but also good direct current (DC) soft magnetic properties (*M_s_* = 63.58 emu/g, *H_c_* = 13.7 Oe). These studies were basically conducted under DC magnetic conditions.

Moreover, soft magnetic materials are commonly used in alternating current (AC) magnetic fields. However, limited information on AC magnetic performance is available in HEAs. Our previous work studied the AC soft magnetic properties of FeCoNi(MnSi)*_x_* HEAs and found that a suitable content of MnSi can improve the AC soft magnetic properties [28]. Duan et al. [29,30] prepared Fe-Co-Ni-Si-Al high-entropy powders by mechanical milling and studied their electromagnetic performance as wave absorbing materials under the frequency ranging from 1 GHz to 16 GHz. It is found that the electromagnetic parameters depend on the milling time and aspect ratio of powders. They also found the wave absorption properties can be improved after annealing in FeCoNiCuAl high-entropy powders [31].

In our work, we chose Fe-Co-Ni-Cu-Al HEAs to study their AC magnetic properties for two reasons. (1) Our previous work showed that FeCoNiCuAl HEA had a combination of good mechanical properties and DC magnetic properties [32]. In particular, it has larger electrical resistivity with 51.4 uΩ⋅cm than 40.8 uΩ⋅cm of non-oriented silicon electrical steel reported in reference [33], which indicates that FeCoNiCuAl HEA has potential AC applications by reducing the energy loss. (2) Recently, Liu et al. [27] found that Ni addition could have obvious effects on the DC magnetic property and mechanical property of FeCoNiCuAl alloy. Here arises the question, does Ni addition have effects on AC soft magnetic properties of Fe-Co-Ni-Cu-Al HEAs? Hence, in this paper, the effects of Ni addition on the AC soft magnetic properties and microstructure in Fe-Co-Ni-Cu-Al HEAs are investigated. The relationship between AC magnetic parameters (e.g., the eddy current loss, *P_e_*, and AC hysteresis loss, *P_h_*) and phase composition is also studied in FeCoNi*_x_*CuAl (1.0 ≤ *x* ≤ 1.75) HEAs. Furthermore, the influencing factors of *P_e_* and *P_h_* are discussed.

## 2. Experimental

The alloy ingots of FeCoNi*_x_*CuAl (1.0 ≤ *x* ≤ 1.75) HEAs were made by arc melting of high purity metals (≥99.99 wt%) with a water-cooled copper crucible, re-melted four times, in a high purity argon atmosphere. The alloys were then sucked into a water-cooled copper mould with the dimensions of 100 × 10 × 2 mm^3^. The composition of samples was confirmed by Electron-coupled plasma atomic emission spectrometry (ICP-AES, PERKINE 7300DV, Perkinelmer, Waltham, MA, USA).

The phases in the alloys were determined by X-ray diffraction (XRD, Rigaku Corporation, Akishima-Shi, Tokyo, Japan) analysis with Cu Kα radiation using D/max-2500 V. The diffraction angles ranged from 20 to 100° and the radiation condition was 18 kW. The microstructure of the alloys was investigated by scanning electron microscope (SEM, TESCAN S9000, Tescan, Brno, Czech Republic) and the chemical compositions of the alloys were measured by energy dispersive spectroscopy (EDS). The AC magnetic characteristic parameters were determined by an AC hysteresis curves test system (FE-2100SM, Yongyi technology co. LTD, Hunan, China) with the sample size of 50 × 9.5 × 1.8 mm^3^. The electrical resistivity (*ρ*) was measured by an ST-2258C multifunction digital four-probe tester.

## 3. Results

### 3.1. XRD

**Figure 1:**
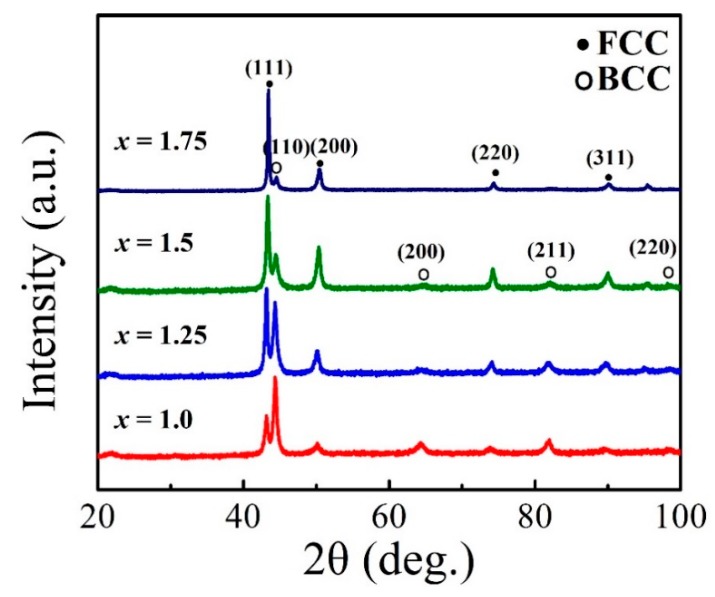
X-ray diffraction (XRD) patterns of the FeCoNi*_x_*CuAl (1.0 ≤ *x* ≤ 1.75) high-entropy alloys (HEAs).

The XRD patterns of the FeCoNi*_x_*CuAl (1.0 ≤ *x* ≤ 1.75) HEAs are shown in Figure 1. All of these HEAs contain two phases, namely FCC and BCC phase. The intensities of the strongest peaks, (111) for FCC and (110) for BCC, are denoted as *I*_(111)F_ and *I*_(110)B_, respectively. Table 1 lists the values of the ratio (*I*_(111)F_*/I*_(110)B_), which is employed to estimate the content of the FCC and BCC phases. It can be seen that the ratio increases with increasing Ni content. It indicates that increasing the Ni content tends to facilitate the formation of the FCC phase in these alloys. The lattice parameters of the FCC and BCC phases were calculated, and the values are also listed in Table 1. With increasing Ni content, the lattice parameters of both phases slightly decrease. This may be due to the atomic radius of Ni (124 pm) being smaller than Fe (126 pm), Co (125 pm), Cu (128 pm) and Al (143.1 pm) [34].

The values of electron concentration (*VEC*), atomic size difference (Δ*R*), electronegativity differences (Δ*X*), mixing entropy (Δ*S*), mixed enthalpy (Δ*H*), and solid solution formation ability (*Ω*) were then calculated and are listed in Table 2. With increasing Ni content, Δ*R,* Δ*X,* Δ*S,* Δ*H* and *Ω* all decrease while *VEC* increases. It is reported that the parameters including *VEC*, Δ*R*, Δ*S* and Δ*H* are key factors to determine the phase formation. In particular, Δ*R* has a critical role in lattice distortion [27]. The decrease of Δ*R* suggests the decrease content of BCC phase [27]. Based on our XRD result and the decrease of the atomic size difference with increasing Ni addition, it may have a conclusion that the increase of Ni content tends to facilitate the formation of the FCC phase in FeCoNi*_x_*CuAl (1.0 ≤ *x* ≤ 1.75) HEAs due to lattice distortion.

### 3.2. SEM Images

**Figure 2:**
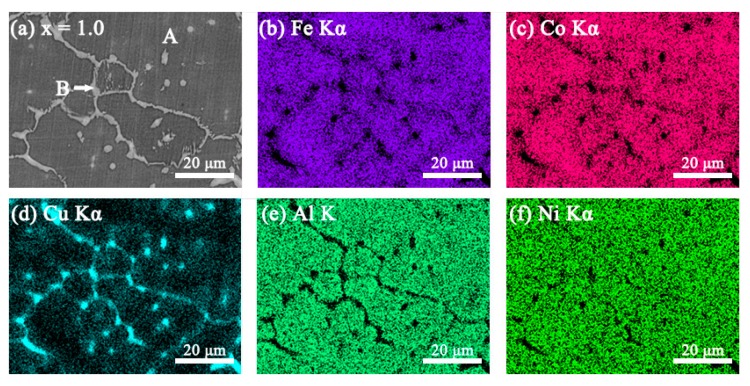
(**a**) SEM-BSE image of FeCoNi_1.0_CuAl high-entropy alloys, and SEM-energy dispersive spectroscopy (EDS) elemental mapping images for (**b**) Fe Kα; (**c**) Co Kα; (**d**) Cu Kα; (**e**) Al K; (**f**) Ni Kα from the same region as (**a**).

**Figure 3:**
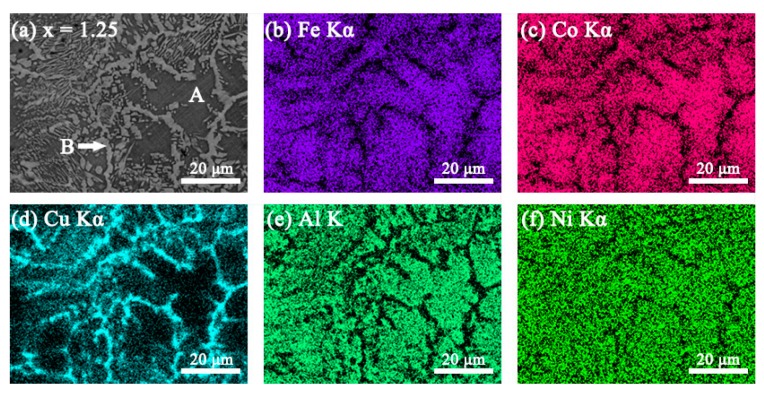
(**a**) SEM-BSE image of FeCoNi_1.25_CuAl high-entropy alloys, and SEM-EDS elemental mapping images for (**b**) Fe Kα; (**c**) Co Kα; (**d**) Cu Kα; (**e**) Al K; (**f**) Ni Kα from the same region as (**a**).

**Figure 4:**
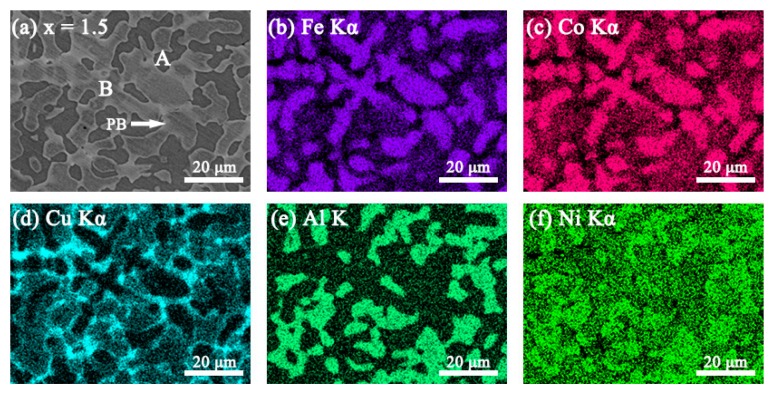
(**a**) SEM-BSE image of FeCoNi_1.5_CuAl high-entropy alloys, and SEM-EDS elemental mapping images for (**b**) Fe Kα; (**c**) Co Kα; (**d**) Cu Kα; (**e**) Al K; (**f**) Ni Kα from the same region as (**a**).

**Figure 5:**
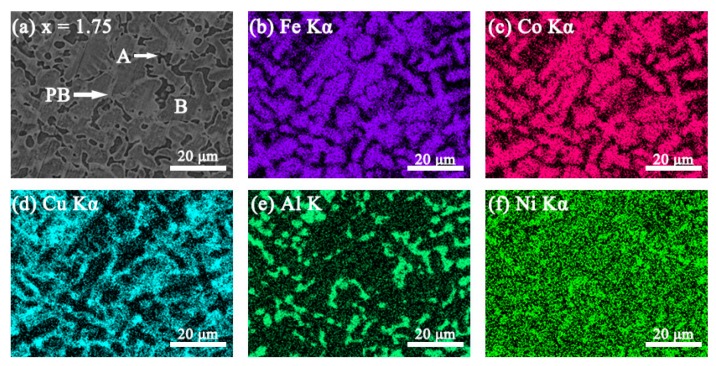
(**a**) SEM-BSE image of FeCoNi_1.75_CuAl high-entropy alloys, and SEM-EDS elemental mapping images for (**b**) Fe Kα; (**c**) Co Kα; (**d**) Cu Kα; (**e**) Al K; (**f**) Ni Kα from the same region as (**a**).

The SEM backscattered electron images (SEM-BSE) and the elemental mapping images of the FeCoNi*_x_*CuAl (1.0 ≤ *x* ≤ 1.75) HEAs are shown in Figure 2, Figure 3, Figure 4 and Figure 5. All the samples show two contrasts: one is dark grey (marked as A), the other is white (marked as B). Combined with XRD result, it is found that region A is BCC phase, and region B is FCC phase. When *x* = 1.0, the elemental mapping images (see Figure 2b–f) show that Cu element is enriched, while Fe, Co, Al and Ni are depleted in FCC phase. In comparison to *x* = 1.0 alloy, the fraction of FCC phase is significantly increased in *x* = 1.25 alloy. The elemental mapping result shows a similar trend of distribution of Cu, Fe, Co, Al and Ni in FCC and BCC phases (see Figure 3b–f). It is worth noting that *x* = 1.5 alloy exhibits different SEM morphology with *x* = 1.0 and *x* = 1.25 alloys, as shown in Figure 4a. Moreover, the elemental distribution in FCC and BCC phases is also different. That is, the mapping images (Figure 4b–f) shows that Al and Ni are depleted whereas Fe and Co are enriched in FCC phase. In particular, a Cu-rich phase boundary (PB) appears between the FCC and BCC phases. This phenomenon of Cu enrichment in the PB region has also been observed in our previous studies [35]. With further increasing Ni content to *x* = 1.75, it consists of a large number of FCC phase and small number of BCC phase. This alloy has similar elemental distribution in FCC and BCC phases to *x* = 1.5 alloy. The EDS point analysis gives the chemical compositions of the alloys in different regions, and it is listed in Table 3. For comparison, the nominal compositions are also included in Table 3.

### 3.3. Magnetic Properties at H = 10 kA/m and f = 50 Hz

**Figure 6:**
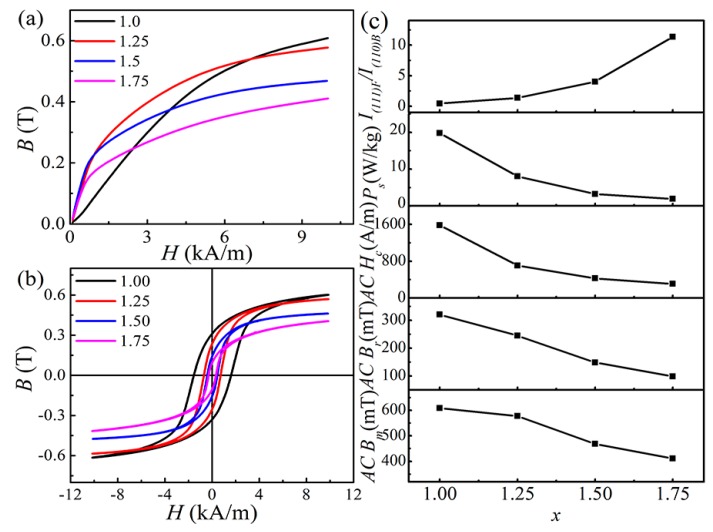
(**a**) The AC magnetization curve; (**b**) The AC hysteresis loops; (**c**) The soft magnetic parameters and *I*_(111)F_*/I*_(110)B_ as a function of *x* for the FeCoNi*_x_*CuAl (1.0 ≤ *x* ≤ 1.75) HEAs measured at *H* = 10 kA/m and *f* = 50 Hz.

Figure 6a,b show the AC magnetisation curves and hysteresis loops of FeCoNi*_x_*CuAl (1.0 ≤ *x* ≤ 1.75) HEAs (*H* = 10 kA/m and *f* = 50 Hz). With increasing Ni content, the magnetisation curve and the hysteresis loop change significantly. The parameters such as the AC maximum magnetic flux density (*B_m_*), AC remanence (*B_r_*), AC coercivity (*H_c_*) and the energy loss (*P_s_*) of the alloy can be obtained, and the detailed values are listed in Table 4. Figure 6c shows *AC B_m_*, *AC B_r_*, *AC H_c_* and *P_s_* of the alloy as a function of Ni content. For comparison, the ratio of *I_(_*_111)F_*/I*_(110)B_ as a function of Ni content is also listed in Figure 6c. With *x* increasing from 1.0 to 1.75, the values of *AC B_m_*, *AC B_r_*, *AC H_c_* and *P_s_* decrease from 608.3 mT to 410.6 mT, 320 mT to 98 mT, 1582 A/m to 306 A/m, and 19.79 W/kg to 1.89 W/kg, respectively. An interesting phenomenon is that the variations of *AC B_m_*, *AC B_r_*, *AC H_c_* and *P_s_* are opposite to that of *I*_(111)F_*/I*_(110)B_. This means that the AC soft magnetic properties of the alloy are closely related to the phase composition. The formation of the FCC phase would be conducive to a decrease in the values of *AC B_r_*, *AC H_c_* and *P_s_*, while it is harmful to *AC B_m_*. This provides a direction for improving AC magnetic properties in the future.

### 3.4. Magnetic Properties at AC B_m_ = 300 mT

**Figure 7:**
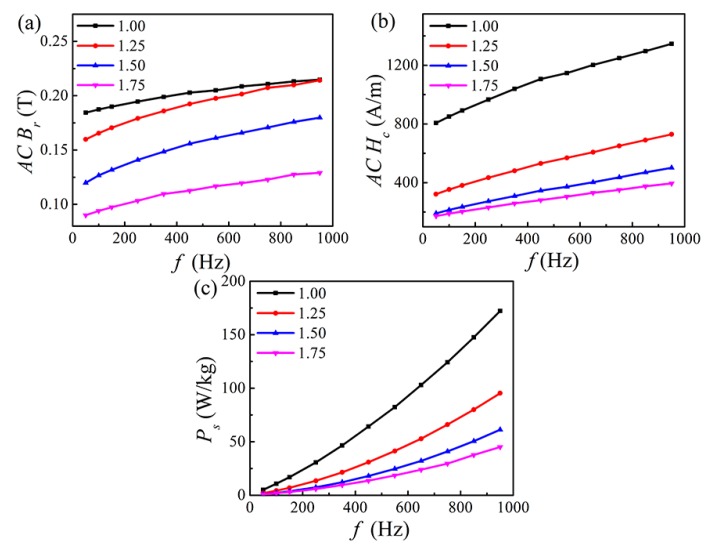
AC soft magnetic parameters as a function of *f* for the FeCoNi*_x_*CuAl (1.0 ≤ *x* ≤ 1.75) HEAs measured at *AC B_m_* = 300 mT (**a**) *AC B_r_*; (**b**) *AC H_c_*; (**c**) *P_s_*.

Soft magnetic materials are usually used at different frequencies, and they need to reach a fixed *AC B_m_* to provide a certain force [36,37]. Figure 7 shows *AC B_r_*, *AC H_c_* and *P_s_* as a function of *f* for the FeCoNi*_x_*CuAl (1.0 ≤ *x* ≤ 1.75) HEAs measured at *AC B_m_* = 300 mT. It can be seen that *AC B_r_*, *AC H_c_* and *P_s_* increase gradually with increasing *f*, and decline with increasing Ni content. When *f* = 950 Hz, with *x* increasing from 1.0 to 1.75, *AC B_r_*, *AC H_c_* and *P_s_* reduce by 40%, 71% and 74%, respectively. The values of *AC B_r_*, *AC H_c_* and *P_s_* are listed in Table 5.

## 4. Discussions

For practical applications of soft magnetic materials, *P_s_* under a dynamic magnetic field is a very important parameter in evaluating the application of magnetic materials. In general, *P_s_* can be decomposed into the sum of three loss generations, *P_h_*, *P_e_* and residual loss (*P_r_*) [38]. Under our test conditions, the effect of *P_r_* can be ignored due to the frequency being not very high [38].

*P_s_* can be expressed as Equation (1) [39]:

(1) $$P_{s}=P_{h}+P_{e}$$

*P_h_* can be expressed as Equation (2) [39]:

(2) $$P_{h}=\eta B_{m}^{n}f$$

where *η* is the material constant, *B_m_* is the maximum magnetic flux density, and *n* is the exponential constant.

*P_e_* can be expressed as Equation (3) [39]:

(3) $$P_{e}=\frac{\pi^{2}d^{2}B_{m}^{2}}{\rho \beta }f^{2}$$

where *d* is the sample thickness, *ρ* is the resistivity, and *β* is the material shape parameter.

Combining Equations (1)–(3) to get Equation (4):

(4) $$P_{s}=P_{h}+P_{e}=\eta B_{m}^{n}f+\frac{\pi^{2}d^{2}B_{m}^{2}}{\rho \beta }f^{2}$$

Setting $a=\frac{\pi^{2}d^{2}B_{m}^{2}}{\rho \beta }$, and $b=\eta B_{m}^{n}$. Then, Equation (4) can be rewritten as Equation (5):

(5) $$\frac{P_{s}}{f}=\frac{P_{h}}{f}+\frac{P_{e}}{f}=b+af$$

Through Equation (5), the unary function of $f_{\left(f,P_{s}/f\right)}$ can be obtained. Using the measured data in Table 5, we can fit the slope a and intercept b of the function. *P_e_* and *P_h_* can then be calculated from *a* and *b*, and the values are listed in Table 5.

Equation (6) can be given by combination of Equations (2) and (5),

(6) $$P_{h}/P_{s}=\frac{1}{1+\frac{a}{b}f}$$

It is seen that *P_h_*/*P_s_* is inversely proportional to frequency. The ratio of *P_h_*/*P_s_* and values of *ρ* are also listed in Table 5.

**Figure 8:**
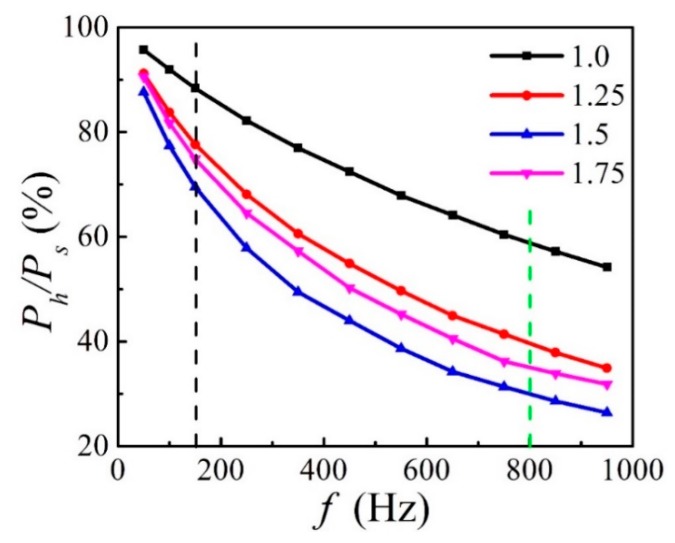
AC hysteresis loss (*P_h_*)*/P_s_* as a function of *f* for the FeCoNi*_x_*CuAl (1.0 ≤ *x* ≤ 1.75) HEAs.

The ratio of *P_h_*/*P_s_* and *P_e_/P_s_* (= 1−*P_h_*/*P_s_*) can be considered as the contribution of *P_h_* and *P_e_* to the total loss, *P_s_*, respectively. Figure 8 shows the *P_h_*/*P_s_* as a function of *f*. With increasing *f*, the ratio of *P_h_*/*P_s_* decreases gradually. It can be seen that when *f* ≤ 150 Hz (left side of black dotted line), *P_h_*/*P_s_* is over 70% which means that *P_h_* mainly contributes to *P_s_*. When *f* ≥ 800 Hz (right side of green dotted line), *P_h_*/*P_s_* is less than 40% (except for the *x* = 1.0 sample) which means that *P_e_* mainly contributes to *P_s_*. Combined with XRD result, it is found that the *P_h_*/*P_s_* decreased with the frequency when Ni addition, x, is less than 1.5 due to an increase of FCC phase. When *x* = 1.5, the ratio of *P_h_*/*P_s_* is only 26.5% at *f* = 950 Hz. However, furthering increasing the fraction of FCC phase results in a minor improvement of *P_h_*/*P_s_*.

**Figure 9:**
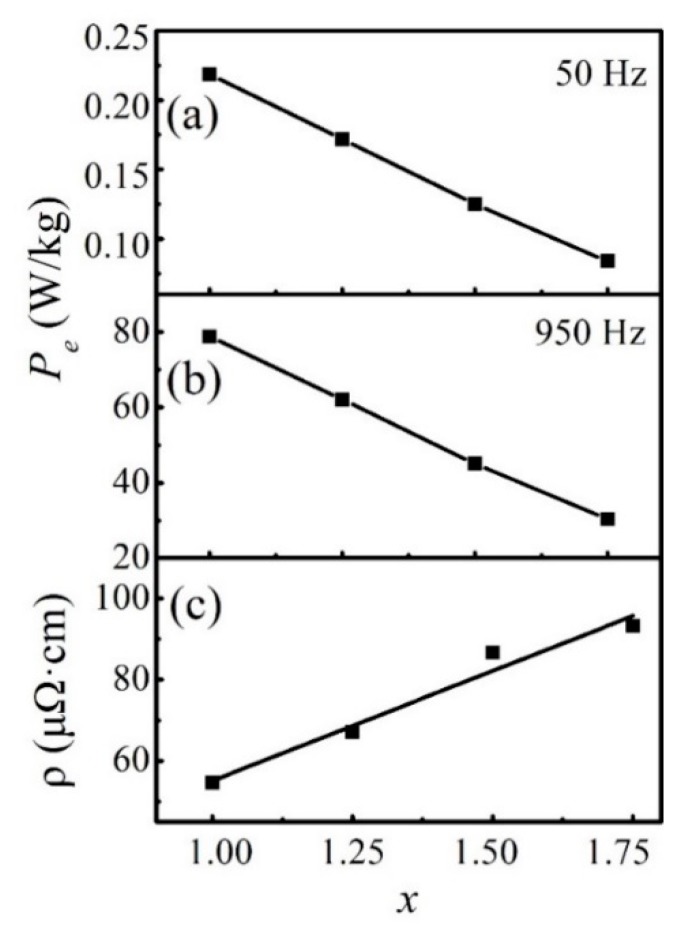
(**a**) Eddy current loss (*P_e_*) at 50 Hz; (**b**) *P_e_* at 950 Hz; (**c**) the *ρ* as a function of *x* for the FeCoNi*_x_*CuAl (1.0 ≤ *x* ≤ 1.75) HEAs.

Figure 9a,b show *P_e_* as a function of *x* for the FeCoNi*_x_*CuAl (1.0 ≤ *x* ≤ 1.75) HEAs at 50 Hz and 950 Hz, respectively, and for comparison purposes, *ρ* as a function of *x* is shown in Figure 9c. With increasing Ni content, *P_e_* decrease linearly, and the downward trend is inversely proportional to the *ρ*, which corresponds to Equation (3). It suggests that *P_e_* can be reduced by increasing *ρ* for the FeCoNi*_x_*CuAl (1.0 ≤ *x* ≤ 1.75) HEAs.

**Figure 10:**
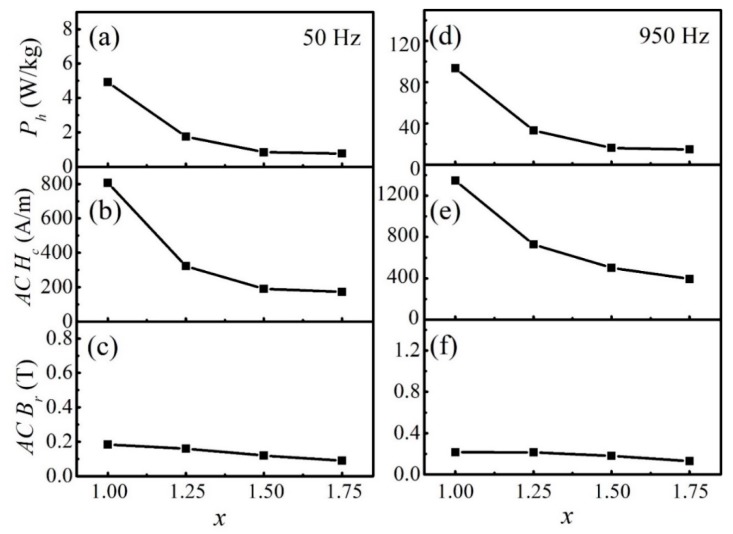
**(a**) *P_h_* at 50 Hz; (**b**) *AC H_c_* at 50 Hz; (**c**) *AC B_r_* at 50 Hz (**d**) *P_h_* at 950 Hz (**e**) *AC H_c_* at 950 Hz (**f**) *AC B_r_* at 950 Hz as a function of *x* for the FeCoNi*_x_*CuAl (1.0 ≤ *x* ≤ 1.75) HEAs.

Figure 10a shows *P_h_* as a function of *x* for the FeCoNi*_x_*CuAl (1.0 ≤ *x* ≤ 1.75) HEAs at 50 Hz. When the Ni content increased from 1.0 to 1.75, *P_h_* was reduced by about 85%. In general, the value of hysteresis loss is related to the area of the hysteresis loop [40], and *B_r_* and *H_c_* are important parameters that determine the area of the hysteresis loop. Figure 10b,c show *AC H_c_* and *AC B_r_* as a function of *x* at 50 Hz, respectively. The scales of *AC H_c_* and *AC B_r_* are in proportion to that of *P_h_*. It can be seen that *AC B_r_* decreases slightly with increasing Ni content. It is worth noting that the downward trend of *AC H_c_* is basically consistent with the downward trend of *P_h_*. This means that the decrease of *P_h_* is closely related to *AC H_c_*. When *f* = 950 Hz, a similar change law can be observed (Figure 10d–f). Therefore, *P_h_* can be reduced by decreasing *AC H_c_* for the FeCoNi*_x_*CuAl (1.0 ≤ *x* ≤ 1.75) HEAs.

Liu et al. used two magnetic parameters (the saturation magnetization, *M_s_*, and the coercivity, *H_c_*) to study the DC magnetic properties of AlCoCuFeNi_x_ (x = 0.5, 0.8, 1.0, 1.5, 2.0, 3.0). They found that high fraction of BCC phase led to high saturation magnetization [27]. Besides *M_s_* and *H_c_*, we used other important soft magnetic parameters including the initial permeability (*μ_i_*), the maximum permeability (*μ_max_*), the remanence (*B_r_*), and the hysteresis loss (*P_u_*) to evaluate DC soft magnetic properties of FeCoNi*_x_*CuAl (1.0 ≤ *x* ≤ 1.75) HEAs. It is shown in Supplementary Table S1. In comparison to Liu’s work, we have minor different values of *M_s_* for *x* = 1.0 and *x* = 1.5 alloys due to the difference of preparation samples.

In our work, the fraction of FCC phase increases with increasing Ni addition. The result of AC magnetic property shows that the increase content of the FCC phase leads to a decrease of *AC B_r_*, *AC H_c_* and *P_s_*, which is beneficial to AC soft magnetic property. The parameters, *B_r_*, *H_c_* and *P_s_*, have close relationships to the microstructure. Our recent work showed the presence of low angle grain boundary with a misorientation angle between 2–5° could reduce dramatically the soft magnetic properties of Fe-Co-Ni-Al alloys [41]. The reason of the decrease of *AC B_r_*, *AC H_c_* and *P_s_* with increasing Ni content might due to a decrease of low angle grain boundary (2–5°) resulting in a release of strain concentration.

It is seen from Table 5 that the electrical resistivity, *ρ*, is 54.7 μΩ·cm for × = 1.00 alloy that is larger than 15 μΩ·cm of mild steel. The values of *ρ* for × ≥ 1.25 alloy are in the range of 67.1−93.3 μΩ·cm, which is larger than 60 μΩ·cm of silicon steel and 50 μΩ·cm of grain-oriented Si steel [42]. It indicates that the FeCoNi*_x_*CuAl (1.0 ≤ *x* ≤ 1.75) HEAs have potential applications in motors, generators and transformers due to relative low energy loss and high electrical resistivity.

## 5. Conclusions

In summary, we study the AC magnetic properties and microstructure of FeCoNi*_x_*CuAl (1.0 ≤ x ≤ 1.75) HEAs. The main results are as follows:

(1) The XRD and SEM results show that the alloys contain two phases, namely a BCC phase and an FCC phase. It is found that increasing the Ni content tends to facilitate the formation of the FCC phase in these alloys due to lattice distortion. When x ≤ 1.25, Cu is enriched in the FCC phase and it is depleted in the BCC phase. When x ≥ 1.5, Cu is depleted in the FCC phase and Al is enriched in the BCC phase. In addition, a Cu-rich phase boundary appears between the FCC and BCC phases.
(2) The formation of the FCC phase would be conducive to a decrease in the values of *AC B_r_*, *AC H_c_* and *P_s_*, while it is harmful to *AC B_m_*. The decrease of *AC B_r_*, *AC H_c_* and *P_s_* with increasing Ni content might due to a decrease of low angle grain boundary (2–5°), resulting in a release of strain concentration. This provides a direction for improvement of the AC magnetic performance in the future.
(3) With increasing *f*, the ratio of *P_h_*/*P_s_* decreases. When *f* ≤ 150 Hz, *P_h_*/*P_s_* is larger than 70%, which means that *P_h_* mainly contributes to *P_s_*. When *f* ≥ 800 Hz, *P_h_*/*P_s_* is less than 40% (except for the *x* = 1.0 sample), which means that *P_e_* mainly contributes to *P_s_*.
(4) At the same frequency, the ratio of *P_h_*/*P_s_* decreases gradually with increasing FCC phase content. *P_e_* is inversely proportional to *ρ*, and *P_h_* is closely related to *AC H_c_*. This provides a direction to reduce *P_s_*.
(5) With increasing Ni content, the value of *ρ* increases from 54.7 μΩ·cm to 93.3 μΩ·cm, which is larger than that of silicon steel. It indicates that the FeCoNixCuAl (1.0 ≤ *x* ≤ 1.75) HEAs have potential applications in motors, generators and transformers due to relative low energy loss and high electrical resistivity.

## Figures and Tables

**Table 1:** The values of *I*_(111)F_*/I*_(110)B_ and lattice parameters of the FeCoNixCuAl (1.0 ≤ x ≤ 1.75) HEAs.

*x*	*I*_(111)F_/*I*_(110)B_	A ± 0.0002 (nm)	
		FCC	BCC
1.0	0.447	0.3618	0.2877
1.25	1.374	0.3614	0.2876
1.5	4.016	0.3610	0.2872
1.75	11.364	0.3608	-

**Table 2:** Parameters of values of electron concentration (*VEC*), atomic size difference (Δ*R*), electronegativity differences (Δ*X*), mixing entropy (Δ*S*), mixed enthalpy (Δ*H*) and solid solution formation ability (*Ω*) for the FeCoNi*_x_*CuAl (1.0 ≤ *x* ≤ 1.75) HEAs.

*x*	*VEC*	Δ*R*	Δ*X*	Δ*S* (J/K·mol)	Δ*H* (kJ/mol)	*Ω* (kJ/mol)
1.0	8.20	5.404	0.1115	1.609R	−5.28	3.8477
1.25	8.29	5.341	0.1102	1.605R	−5.51	3.6980
1.5	8.36	5.278	0.1090	1.594R	−5.69	3.5831
1.75	8.43	5.216	0.1077	1.579R	−5.81	3.4932

**Table 3:** The chemical compositions (at.%) of the FeCoNi*_x_*CuAl (1.0 ≤ *x* ≤ 1.75) HEAs by EDS point analysis.

*x*	Regions	Fe	Co	Ni	Cu	Al
1.0	Nominal	20.00	20.00	20.00	20.00	20.00
	A	19.74	20.00	20.06	18.98	21.23
	B	15.54	15.12	17.27	37.01	15.07
1.25	Nominal	19.05	19.05	23.80	19.05	19.05
	A	21.31	21.69	23.92	12.34	20.74
	B	15.77	15.05	21.61	32.39	15.18
1.5	Nominal	18.18	18.18	27.28	18.18	18.18
	A	13.56	15.50	28.33	18.56	24.05
	B	21.93	22.20	26.96	13.01	15.90
	PB	13.21	13.69	24.21	32.72	16.17
1.75	Nominal	17.39	17.39	30.44	17.39	17.39
	A	12.37	13.68	31.71	17.75	24.49
	B	20.93	20.58	30.04	13.4	15.07
	PB	13.13	13.52	29.95	28.10	15.29

**Table 4:** AC maximum magnetic flux density (*B_m_*), AC remanence (*B_r_*), AC coercivity (*H_c_*) and energy loss (*P_s_*) of the FeCoNi*_x_*CuAl (1.0 ≤ *x* ≤ 1.75) HEAs measured at *H* = 10 kA/m and *f* = 50 Hz.

*x*	*AC B_m_* (mT)	*AC B_r_* (mT)	*AC H_c_* (A/m)	*P_s_* (W/kg)
1.0	608.3	320.0	1582.0	19.79
1.25	577.3	244.9	705.3	8.02
1.5	468.2	148.2	424.7	3.24
1.75	410.6	98.2	306.3	1.89

**Table 5:** AC magnetic parameters measured at *AC B_m_* = 0.3 T at different *f* and electrical resistivity of the FeCoNi*_x_*CuAl (1.0 ≤ *x* ≤ 1.75) HEAs.

*x*	*F* (Hz)	*P_s_* (W/kg)	*P_h_* (W/kg)	*P_e_* (W/kg)	*P_h_*/*P_s_* (%)	*AC H_c_* (A/m)	*AC B_r_* (mT)	*Ρ* (μΩ·cm)
1.0	50	5.13	4.91	0.22	95.7	806.7	184.4	54.7
1.25		1.93	1.76	0.17	91.2	322.2	159.8	67.1
1.5		0.97	0.85	0.12	87.6	190.5	119.9	86.7
1.75		0.85	0.77	0.08	90.6	172.7	90.1	93.3
1.0	100	10.78	9.91	0.87	91.9	850.2	187.4	54.7
1.25		4.25	3.56	0.69	83.8	353.3	165.5	67.1
1.5		2.21	1.71	0.50	77.4	214.7	126.7	86.7
1.75		1.86	1.52	0.34	81.7	189.0	94.0	93.3
1.0	150	16.91	14.94	1.97	88.4	891.9	189.9	54.7
1.25		6.94	5.39	1.55	77.7	381.6	170.5	67.1
1.5		3.68	2.56	1.12	69.5	235.4	131.8	86.7
1.75		3.02	2.26	0.76	74.9	203.5	97.4	93.3
1.0	250	30.71	25.25	5.46	82.2	966.5	194.6	54.7
1.25		13.49	9.19	4.30	68.1	434.3	179.1	67.1
1.5		7.40	4.28	3.12	57.9	273.1	140.9	86.7
1.75		5.92	3.82	2.10	64.5	231.5	103.4	93.3
1.0	350	46.49	35.78	10.71	77.0	1039	198.9	54.7
1.25		21.40	12.97	8.43	60.6	481.5	185.9	67.1
1.5		12.11	5.99	6.12	49.5	308.7	148.5	86.7
1.75		9.63	5.52	4.11	57.3	258.6	109.6	93.3
1.0	450	64.18	46.48	17.70	72.4	1106	202.8	54.7
1.25		30.89	16.96	13.93	54.9	531.3	192.4	67.1
1.5		18.05	7.94	10.11	44.0	345.7	155.9	86.7
1.75		13.67	6.87	6.80	50.3	281.2	112.6	93.3
1.0	550	82.32	55.88	26.44	67.9	1147	205.0	54.7
1.25		41.35	20.55	20.80	49.7	569.1	197.6	67.1
1.5		24.63	9.52	15.11	38.7	372.2	161.1	86.7
1.75		18.55	8.39	10.16	45.2	305.0	116.8	93.3
1.0	650	103.00	66.08	36.92	64.2	1202	208.6	54.7
1.25		52.79	23.73	29.06	45.0	607.5	201.5	67.1
1.5		32.10	11.00	21.10	34.3	402.8	165.9	86.7
1.75		23.87	9.68	14.19	40.6	330.8	119.5	93.3
1.0	750	124.30	75.14	49.16	60.5	1249	210.7	54.7
1.25		66.04	27.35	38.69	41.4	650.9	207.3	67.1
1.5		40.93	12.84	28.09	31.4	436.4	170.8	86.7
1.75		29.61	10.72	18.89	36.2	350.7	122.8	93.3
1.0	850	147.50	84.36	63.14	57.2	1296	213.1	54.7
1.25		80.00	30.31	49.69	37.9	690.5	209.9	67.1
1.5		50.54	14.46	36.08	28.6	470.4	175.9	86.7
1.75		37.63	13.37	24.26	35.5	375.0	127.5	93.3
1.0	950	172.20	93.33	78.87	54.2	1346	214.8	54.7
1.25		95.37	33.30	62.07	34.9	729.4	214.2	67.1
1.5		61.25	16.18	45.07	26.4	501.5	179.8	86.7
1.75		45.13	14.83	30.30	32.9	395.5	129.0	93.3

## Supplementary Materials

The following are available online at https://www.mdpi.com/1996-1944/12/24/4222/s1, Table S1: Direct current (DC) soft magnetic properties of FeCoNi_x_CuAl (1.0 ≤ x ≤ 1.75) high-entropy alloys.
