# Supplementary material for: The AC Soft Magnetic Properties of FeCoNixCuAl (1.0 ≤ x ≤ 1.75) High-Entropy Alloys
Source: Materials (Basel). 2019 Dec 16;12(24):4222. doi: 10.3390/ma12244222 (PMC6947456; doi:10.3390/ma12244222)
Supplement: Supplementary file 1 [file materials-12-04222-s001.pdf]

materials

Article

The AC Soft Magnetic Properties of $FeCoNi_xCuAl$ ($1.0 \leq x \leq 1.75$) High-Entropy Alloys

Zhongyuan Wu [1], Chenxu Wang [1], Yin Zhang [1], Xiaomeng Feng [1], Yong Gu [1,2], Zhong Li [3], Huisheng Jiao [4], Xiaohua Tan [1,*] and Hui Xu [1,*]

1 Institute of Materials, School of Materials Science and Engineering, Shanghai University, Shanghai 200072, China; wzy951122@gmail.com (Z.W.); wcx123581@163.com (C.W.); zhangyin0208@163.com (Y.Z.); fengxiaomeng96@163.com (X.F.); gy2018@hznu.edu.cn (Y.G.)
2 Qianjiang College, Hangzhou Normal University, Hangzhou 310036, China
3 College of Materials and Environmental Engineering, Institute for Advanced Magnetic Materials, Hangzhou Dianzi University, Hangzhou 310018, China; hanying880205@163.com
4 Tescan China, Ltd., Shanghai 201112, China; Huisheng.Jiao@tescanchina.com
* Correspondence: tanxiaohua123@163.com (X.T.); Huixu8888@shu.edu.cn (H.X.); Tel.: +86-021-563-378-87 (H.X.)

Table S1. Direct current (DC) soft magnetic properties of $FeCoNi_xCuAl$ ($1.0 \leq x \leq 1.75$) high-entropy alloys.

x	M_s (Am^2/kg)	μ_i	μ_m	P_u (J/m^3)	B_r (mT)	H_c (A/m)
1.00	78.69	45.6	150.0	2019	293.6	998.8
1.25	75.60	132.4	300.8	737.2	235.3	383.0
1.50	59.33	213.4	383.3	241.1	127.8	178.3
1.75	54.44	181.1	289.4	205.2	88.7	167.8

© 2019 by the authors. Submitted for possible open access publication under the terms and conditions of the Creative Commons Attribution (CC BY) license (http://creativecommons.org/licenses/by/4.0/).
